# Evaluation of oral care using MA-T gel for high-risk patients: a pilot study

**DOI:** 10.1186/s12903-023-02779-5

**Published:** 2023-02-17

**Authors:** Hitomi Ono-Minagi, Nao Gojo, Tsutomu Nohno, Tsuyoshi Inoue, Hideyo Ohuchi, Takayoshi Sakai

**Affiliations:** 1 Department of Cytology and Histology, Okayama University Graduate School of Medicine, Dentistry and Pharmaceutical Sciences, 2-5-1, Shikata-Cho, Okayama, 700-8558 Japan; 2 Division of Hospital Dentistry, Central Clinical Department, Okayama University Hospital, Okayama, Japan; 3 Research Fellow of Japan Society for the Promotion of Science, Tokyo, Japan; 4 Department of Oral-Facial Disorders, Osaka University Graduate School of Dentistry, 1-8, Yamadaoka, Suita, Osaka, 565-0871 Japan; 5 Department of Cytology and Histology, Okayama University Medical School, Okayama, Japan; 6 Institute for Open and Transdisciplinary Research Initiatives, Osaka University, Osaka, Japan; 7 Division of Advance Pharmaco-Science, Osaka University Graduate School of Pharmaceutical Sciences, Osaka, Japan; 8 Department of Cytology and Histology, Faculty of Medicine, Dentistry and Pharmaceutical Sciences, Okayama University, Okayama, Japan

**Keywords:** Bacteria, Oral hygiene, Xerostomia, Opportunistic infection, Infection control, Dysphagia

## Abstract

**Background:**

Oral care with gel is a common method for preventing aspiration in high-risk patients. An oral care gel is used to clean and moisturize the oral cavity. However, the effects of gel care on the oral bacteria remain unclear. In this pilot study, we described a matching transformation system (MA-T) for elderly high-risk patients. MA-T is an on-demand aqueous chlorine dioxide solution that provides excellent safety and has various antimicrobial activities, even in the presence of abundant organic compounds. This study investigated the effects of MA-T gel in patients requiring nursing care.

**Materials and methods:**

Patients who were hospitalized for nursing care were included in this study. No drugs and foods were administered orally. Oral bacteria and intraoral humidity were examined by daily care using MA-T gel. Moreover, oral membranous substances were analyzed and material from the oral cavity was cultured on selective media for identifying opportunistic organisms.

**Results:**

Membranous substances were present in the oral cavities of all patients. The number of bacteria decreased, and oral moisture improved, after treatment with MA-T gel. Moreover, oral humidity was also controlled with the continued use of MA-T gel. MA-T gels should be used not only for professional care but also on a daily basis for better oral care. Furthermore, the results of bacterial cultures showed that MA-T controls the propagation of opportunistic bacterial infections.

**Conclusion:**

Membranous substances may be observed in the oral cavity of individuals requiring nursing care for tube feeding. The results of this pilot study suggest that MA-T, a novel disinfectant, can be used for oral care in the elderly to reduce the risk of aspiration-pneumonia.

**Supplementary Information:**

The online version contains supplementary material available at 10.1186/s12903-023-02779-5.

## Background

Dysphagia is a symptom of swallowing dysfunction that is caused by a variety of conditions such as cerebrovascular disorder [1]. Dysphagia was observed in 55% of elderly patients admitted with care-associated pneumonia (CAP) [2]. Loeb et al. [3] conducted a case–control study and demonstrated that dysphagia was observed in 14.3% of elderly patients hospitalized for pneumonia. Oral hygiene is critical for the prevention of aspiration. Oral bacteria may be an important reservoir for CAP in institutionalized elders who are on parenteral nutrition and are at high risk for developing aspiration pneumonia [4]. Although oral care is important for the prevention of aspiration pneumonia, there are many cases in which it is difficult to clean smooth tissues or teeth surfaces. The oral cavities of patients on parenteral nutrition are dry, and their care is time-consuming due to the risk of bleeding. Moreover, special care has to be taken because of the risk of aspiration. Such patients are more likely to develop severe diseases or complications after infection. Adequate oral care using a safe method is important for preventing dysphagia and reducing the risk of aspiration. Oral care for the elderly is different from that for healthy people, and it is necessary to manage oral hygiene, including moisturizing, in addition to brushing the teeth. In these cases, membranous substances may be observed in the oral cavity of patients receiving Ryle’s tube-feeding. All membranous substances were composed of keratinous material derived from the stratified squamous epithelium of the oral mucosa. It has been reported that parenteral intake and dryness of the oral mucosa are important factors for its formation [5]. Despite the fact that a lot of bacteria are present in membranous substances, it is not clear whether intraoral bacteria decrease on temporarily cleaning membranous substances.

A common method of oral care in high-risk patients is using gels [6]. Oral care gels are also effective in patients with xerostomia [7]. In practice, xerostomia can impair swallowing, speech, and oral hygiene; if left unchecked, symptoms such as dysphagia and dysarthria can diminish patients’ quality of life (QOL). Xerostomia is one of the factors contributing to dysphagia [8]. The principal idea of these salivary substitutes is to provide a long-continuous coat over oral soft tissues using an oral care gel [9]. Xerostomia is often observed in hospitalized or older adults requiring long-term care [10]. Tube-feeding and mechanical airways decrease the auto-purification of saliva. In cases of xerostomia, membranous substances observed on the tongue and palate are risk factors for halitosis and aspiration pneumonia.

In this study, we evaluated the effectiveness of oral care gel using a matching transformation system (MA-T). MA-T is an on-demand aqueous chlorine dioxide solution with the generation of aqueous radicals controlled by a catalyst [11]. MA-T exhibits excellent safety and various antimicrobial effects [12]. MA-T showed sufficient antimicrobial effects at 100 ppm in all the media. Additionally, MA-T is not inflammable, volatile, or corrosive; therefore, it is widely used in medical care. In this study, we focused on the effects of MA-T in high-risk patients. Based on the hypothesis that MA-T is effective for improving oral hygiene, we prospectively tested its efficacy against these pathogens.

This study aimed to investigate the effect of MA-T gel on patients who were on parenteral nutrition. The effectiveness of oral care provided by the gel was examined at the bedside and in the laboratory side by assessing oral bacterial counts and oral moisture.

## Methods

### Study design

This interventional study assessed the efficacy of MA-T gel. Patients included in the study were hospitalized in Okayama Prefecture, Japan. This study was conducted between April and June 2022. Patients who underwent medical examination and treatment and were put on parenteral nutrition were included in the study. The exclusion criteria were as follows: patients who could not provide consent and those with a relatively good oral environment. The oral environment was assessed using the Oral Assessment Guide score [13]. A flowchart of the patient selection process is shown in Additional file 1: Figure S1. All patients required nursing care and underwent medical examination and treatment. The patients were managed by tube feeding and assisted respiration using a mechanical ventilator. They had been receiving nasal feeding for at least 1 year. They were followed up for several years, so their dental treatment had been completed, and they continued to receive professional care prior to the study. Five participants were included in the study. However, two patients died during this study, as a consequence of a critical underlying disease. Three patients were included in the study**.** All sampling was repeated three times using the same method. None of the patients developed severe pneumonia during the study period. Informed consent was obtained from the relatives for inclusion of the patients in the study. The clinical characteristics of the patients are presented in Table 1.

**Table 1:** Participant characteristics

Patient	Age	Sex	Nutritional intake	Type of ventilation	Primary disease
A	64	Female	NG	TR	Cerebral hemorrhage
B	71	Male	NG	TR	Cerebral infarction Diabetes
C	87	male	NG	NPPV	Cerebral hemorrhage Cerebral infarction Diabetes Chronic hepatitis

*NG* nasogastric intubation ventilation, *TR* tracheostomy, *NPPV* noninvasive positive-pressure ventilation

### Study protocol for the research participants

The two phases of MA-T gel and control were compared in the same patient during the investigation period. Pure water was used as a control instead of the MA-T gel. Continuous professional care was provided during the investigation period. The first intervention was performed with pure water and without any oral care products. The next period of investigation involved care with MA-T gel. During the MA-T gel investigation, the gel was placed at the bedside and used for daily care. Before each investigation period, a month reset periods was set up. During the reset period, the patients did not receive professional care. Daily care was consistently provided throughout the reset and study period. Daily care was done using the same type of oral swab sponge and toothbrush, and that was provided by ward nurses three times a day, between 6:00 and 10:00, 12:00 and 16:00, and 18:00 and 20:00. During investigation periods, daily care was not provided at noon. Instead, dental hygienists with more than 20 years of experience in dentistry cleaned the oral cavity with 2.0 g pure water or MA-T gel. The duration of the oral care was not limited. Because the patient’s position was limited owing to contractures and pressure ulcers, oral care was provided keeping the patient’s comfort in mind. The same types of new oral swab sponges, toothbrushes, tufts, and interdental brushes were used. The following basic information was extracted from medical records: age, sex, nutritional intake, type of ventilation, and primary disease. In all cases, physical conditions were checked before the investigation. Body temperature was measured using a thermometer, and blood oxygen saturation was measured using a pulse oximeter. In addition, blood pressure was measured to check for any changes in the daily status.

### Oral care gel

MA-T gel; N.act oral removal gel^®^ is available from Earth Corporation (Tokyo, Japan). The chief ingredient of MA-T gel is chlorous acid ion. Other ingredients included solvent, water, viscosity conditioner, hydroxypropyl methylcellulose, flavoring materials, xylitol, pH conditioner, sodium phosphate, disodium phosphate, and sodium hydroxide; cleaning materials, sodium chlorite; and preservative, benzalkonium chloride. The mouth gel contained 100 ppm MA-T. The control gel for the cultural experience was provided by the Earth Corporation. The control gel consisted of a base material in the MA-T gel and did not contain MA-T. Although water was used as a control in the patient surveys, we used similar quantities of water and the MA-T gel. MA-T mouthwash; A2Care mouthwash^®^ (Earth Corporation and a2care Corporation, Tokyo, Japan) has been commercially available without any adverse events for 7 years since 2016. There have been no reports of harmful effects caused by the use of MA-T gels since the beginning of 2022.

### Study methods

The research was conducted at the bedside and laboratory-side. Bacterial count measurements, oral moisture tests, and opportunistic bacterial count measurements were performed in the bedside study. Histological and bacterial culture analyses were performed in the laboratory.

### Bacterial count measurement

Bacterial counts were performed using an oral bacterial counter^Ⓡ^ (Panasonic, Tokyo, Japan). The bacteria counter measures all the living bacteria. It collects bacteria in the liquid by dielectrophoretic on the electrode, measures the impedance change, and converts it into the bacterial concentration (CFU) in 1.0 mL of the sample, thus quantitatively measuring the number of bacteria. Samples were collected from the oral mucosa using cotton swabs. The number of bacteria is affected by intraoral humidity [14]. The oral cavity was wiped using a sponge brush soaked in a small amount of water before professional care. The samples were measured before swabbing, after swabbing, and after professional care a total of three times.

### Oral moisture test using Mucus^®^

Moisture levels of the oral mucosa were measured using an oral moisture-checking device (Mucus^®^, LIFE Co., Saitama, Japan). The device analyzes water maintenance of the mucous membrane [15]. The oral moisture score of the buccal mucosa was repeated thrice, and the median value was selected. Scores ≤ 27.9 were categorized as xerostomia. Scores between 28.0 and 29.5 are considered borderline between normal and xerostomia, and a score ≥ 29.6 is considered normal.

### Opportunistic bacterial count measurement

Opportunistic bacterial infections were corrected prior to swabbing and professional care. All opportunistic bacterial counts were measured by BML, Inc. (Tokyo, Japan). Nutrient medium with the samples was cultured at 35.0 ± 1.0°C for 18–24 h under 5.0% CO_2_. Colonies of the desired bacteria were identified using a confirmation nutrient medium and an identification kit. For opportunistic infection analysis, we assessed 10 types of pathogens: *methicillin-resistant Staphylococcus aureus* (*MRSA*), *methicillin-sensitive Staphylococcus aureus (MSSA)*, *Haemophilus influenzae (H. influenzaw)*, *Streptococcus agalactiae (S. agalactiae)*, *Pseudomonas aeruginosa (P. aeruginosa)*, *Streptococcus pneumoniae (S. pneumoniae)*, *Klebsiella pneumoniae (K. pneumoniae)*, *Serratia marcescens (S. marcescens)*, *Moraxella catarrhalis (M. catarrhalis)*, and *Candida species.* The opportunistic infection bacterial score was set as follows: (—): No growth of bacteria, (1+): The growth of bacteria accepted to be one-third of the nutrient medium (approximately 1.0 × 10^3^ CFU/mL), (2+): The growth of bacteria accepted to be two-thirds of the nutrient medium (approximately 1.0 × 10^4^ CFU/mL), (3+): The growth of bacteria accepted by the whole nutrient medium (above 1.0 × 10^5^ CFU/mL).

### Histological analysis

Histological analysis was performed to confirm the structures of the membranes. The membranes used in the laboratory study were samples obtained after the reset period and before the intervention began. Samples were obtained from the patients’ palates by dental hygienists. The membranes were blindly selected as a sample of middle softness and sectioned by the technical staff. After intraoral photographs were taken, the adherent material of the palate was collected and immersed in 4.0% neutral buffered formalin solution. Paraffin sections of the collected membranous material were prepared according to the standard method, and hematoxylin and eosin staining was performed [16].

### Bacterial culture analysis

The control gel and MA-T gel were used to compare reactivity to bacteria. Most gram-positive and gram-negative bacteria were cultured on cetrimide agar (Nissui, Tokyo, Japan). *MRSA* and *P*. *aeruginosa*-selective media were used for culture of the respective organisms. Oral film of 0.5 g and 1.0 mL of sterilized distilled pure water were mixed by vortex for 1 min after being left for 10 min at 28 °C. Half of the liquid part of the mixed oral epithelium was mixed with gel and spread in the culture medium prepared in 9-cm diameter plates. Three experiments were performed. After culturing the cells for 24 h, the number of bacteria was evaluated.

### Ethical approval and consent

The Institutional Ethical Review Board of Osaka University Graduate School of Dentistry approved the study protocol (Approval Number: R2-E40-4). All methods were performed in accordance with relevant guidelines and regulations. Informed consent was provided by the patients’ families because all the patients were unable to communicate. We confirmed that informed consent described the conditions of donation of materials for this research, such as oral membranous substances.

### Statistical analysis

Data are presented as mean values and standard deviations. We used the paired *t* test to compare the MA-T gel and water. The Bonferroni correction was used for multiple comparisons (Before, Swab, and Care). Spearman rank correlation coefficients were used to determine the relationship between the moisture score and bacterial count. Fisher’s exact test was used for opportunistic infection bacterial score analyses.

## Results

### Patient demographics

This study consisted of clinical surveys from the bedside to basic laboratory research. The aim of this study was to identify the efficacy of MA-T for high-risk patients. The mean age of the patients was 74.0 ± 6.8 years (minimum 64 and maximum 87 years).

None of the patients could not communicate because of past cerebrovascular accidents. They were unable to ingest orally and were fed by Ryle’s tube. During the investigation period, none of the patients had a fever or other symptoms suggestive of pneumonia.

### Study design

**Fig. 1:**
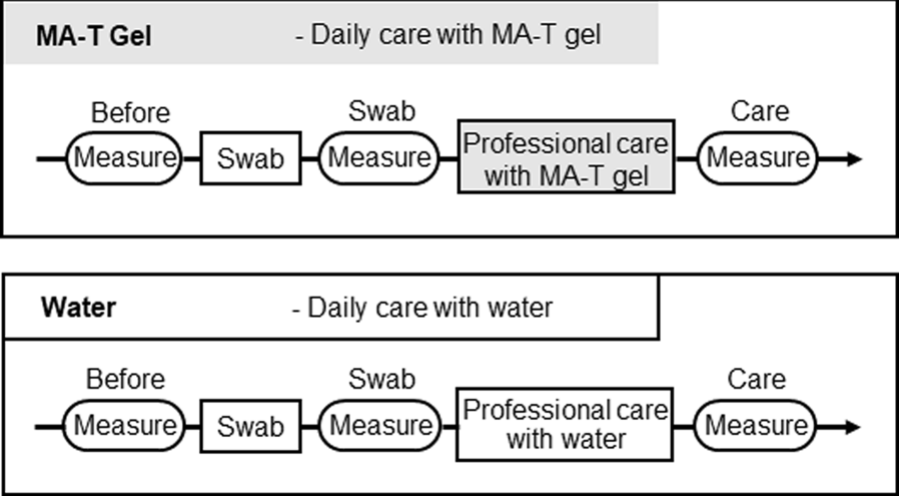
Flow diagram. The patients received daily oral care during the following week. Matching transformation system (MA-T) gel: Oral care is provided daily by nurses using MA-T gel. Control: Oral care with water was provided daily. Daily oral care included tooth brushing twice daily by the nurses. Dental hygienists provided professional oral care. First, the total oral bacterial count, opportunistic bacterial count, and oral moisture score were measured (before). The oral cavity was gently swabbed with a sponge brush soaked in water. After swabbing, the total oral bacterial count and oral moisture score were measured (swabs). Furthermore, MA-T gel or water is applied to the lips, tongue, palate, and buccal mucosa once and cleaned using tufts and interdental brushes. After assisted brushing of the teeth, membranous material was removed using tweezers and gauze. Finally, the total oral bacterial count and oral moisture score were measured (Care). The MA-T gel was applied to the tongue and buccal mucosa. Thus, professional oral care consists of “assisted tooth brushing + removal of exfoliated epithelial film + application/no application of MA-T gel.”

The oral environment of the patients in this study was dry, and membranous substances were attached to their oral cavities. No change was detected in the number of bacteria before and after swabbing using a bacterial counter (Fig. 1). However, there were no differences in the preliminary research. The black triangle represents the single administration data that carried out daily care with water, and MA-T gel was used for professional care. (Additional file 2: Figure S2A, B, C).

### Oral condition and care

**Fig. 2:**
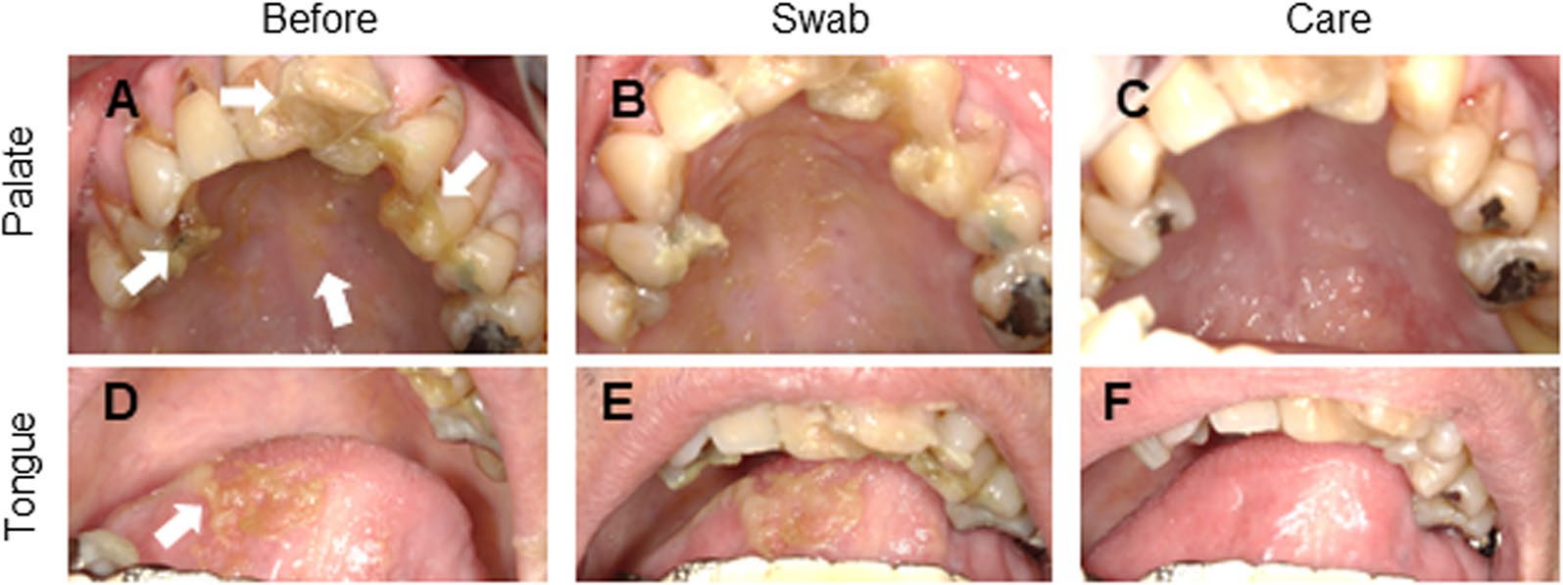
Intraoral photographs during oral care. Upper occlusal view (**A**–**C**), Intra-frontal view of the tongue (**D**–**F**). “Before” means before treatment (**A**, **D**). White arrows indicate the exfoliated epithelial membranes. “Swab" means after swabbing with a sponge brush (**B**, **E**), and “Care” means after professional care (**C**, **F**)

The palatal area was the most common site of membranous substances in both groups. The back of the tongue was the second most common site. Membranous substances often adhere to the oral mucosa and cannot be easily eliminated using a sponge brush (Fig. 2A, B, D, E). Dental hygienists performed and completed professional care when all visually observed debris was eliminated (Fig. 2C, F). There was no significant difference in the care time between the usual water care and MA-T care groups (data not shown). The number of bacteria was compared before and after professional care.

### Effect of MA-T gel on patients

**Fig. 3:**
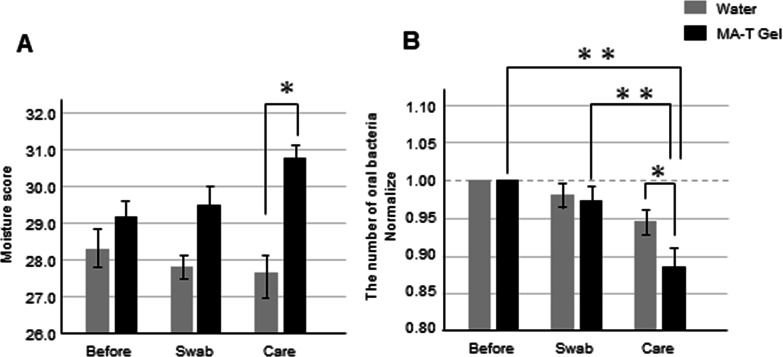
Difference in counts of oral moisture and total bacteria between water care and MA-T gel care groups. The bar graphs show oral moisture sampling data from three participants, three times each. *P < 0.05. Changes in oral moisture during oral care, between care with water and using moisture gels (**A**). Changes in the number of bacteria during oral care with water and moisture gels. Data were normalized based on prior information among the individuals. *P < 0.05; **P < 0.01 (**B**)

**Fig. 4:**
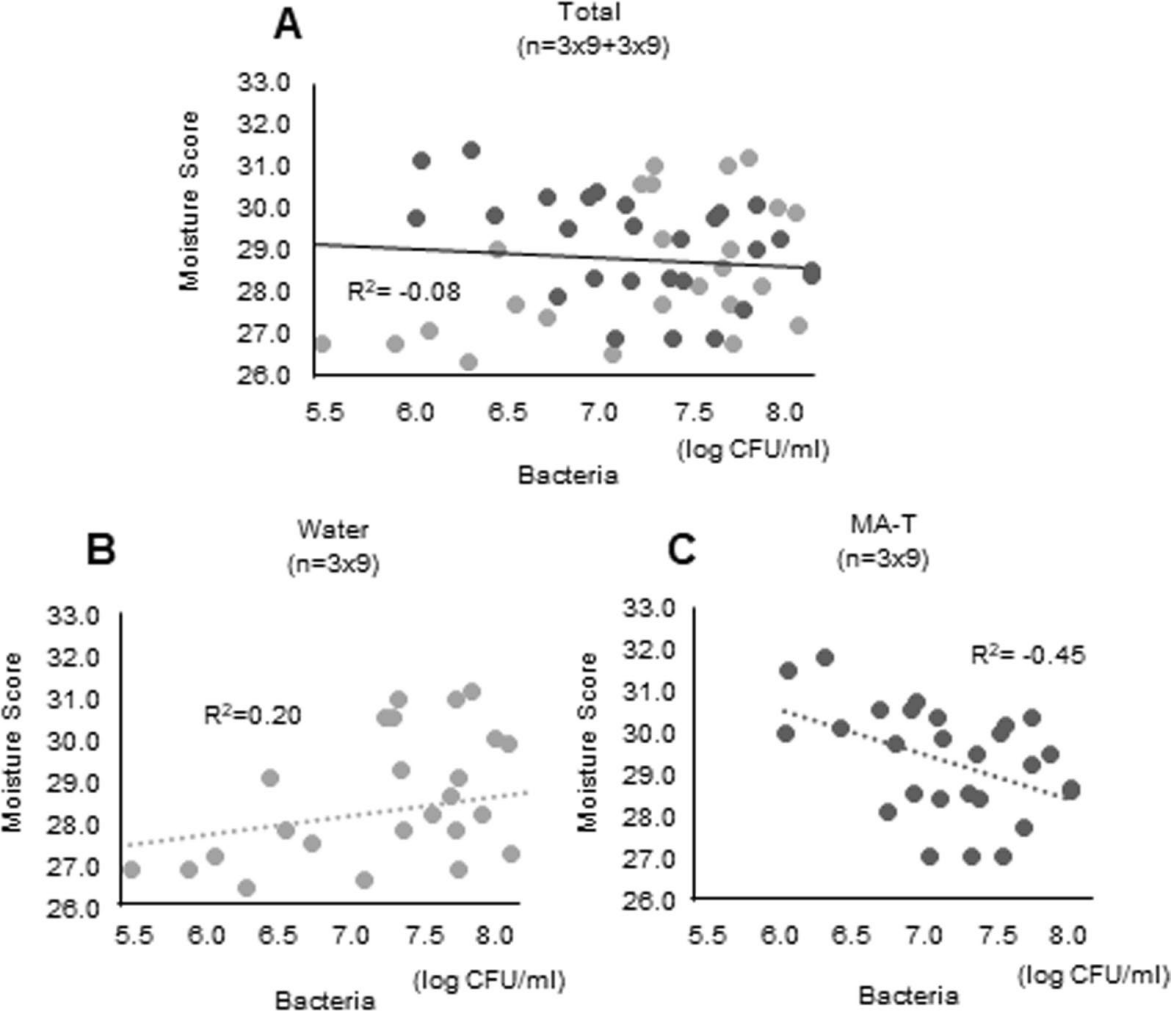
Correlation between the scores of oral bacteria and oral moisture. The total indicates the results of sampling data from three participants, three times each (n = 54) (**A**). The graph shows the results of sampling data, when using water for daily care three times (n = 27) (**B**). Daily care using MA-T gels (n = 27) (**C**)

We evaluated the differences in oral wetness and total bacterial count between water and MA-T gel care. The moisture content of oral mucous membranes was measured using a sensor device. When MA-T gel was used for daily care, high oral moisture levels were noted. After professional care, the moisture content was higher for those treated using the MA-T gel than for those treated with water (Fig. 3A). Additionally, we investigated the effects on the bacterial count. The bacterial count was normal before swabbing. Before professional care, there was no significant difference between the two groups; however, the total bacterial count was significantly reduced after professional care (Fig. 3B, non-normalized data in Additional file 3: Figure S3). When only water was used for oral care, the number of bacteria before and after treatment was similar. However, the number of bacteria decreased by one-tenth or less when performing daily care using the gel. Furthermore, the relationship between intraoral humidity and the number of bacteria was important in this study and was extensively analyzed. In all sampling data, the intraoral humidity and number of bacteria did not correlate (R^2^ = − 0.08) (Fig. 4A). The oral moisture score and the number of bacteria showed a mild positive correlation when using water (R^2^ = 0.20) (Fig. 4B). However, the oral moisture score and the number of bacteria during MA-T care showed a negative correlation, contrary to the results with water care (R^2^ = − 0.45) (Fig. 4C).

### Effect of MA-T gel on opportunistic bacteria

**Fig. 5:**
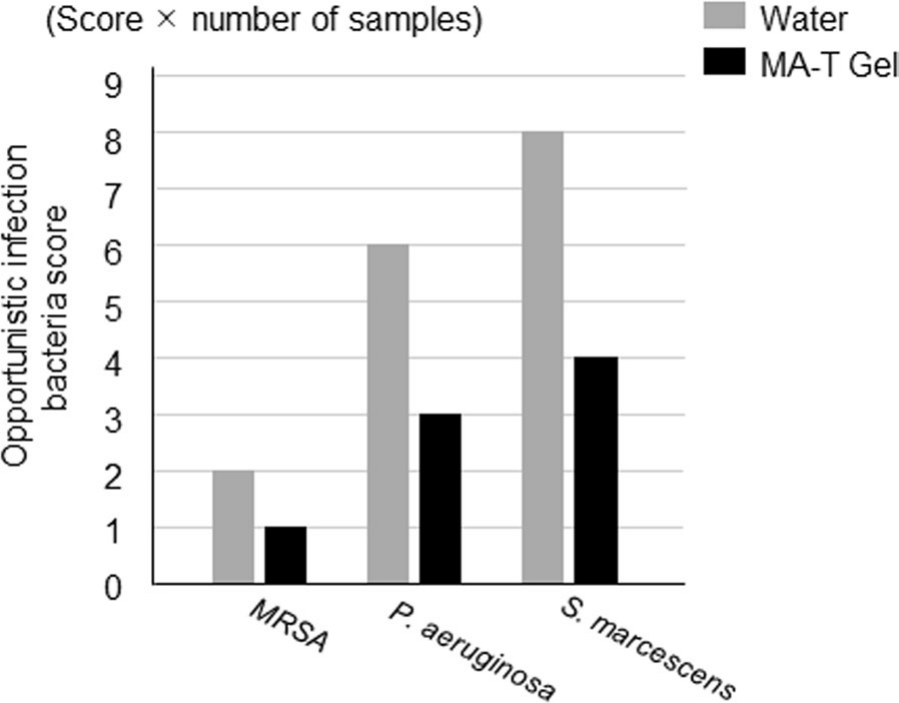
Differences in opportunistic bacterial counts between water and MA-T gel groups. The opportunistic infection bacteria score multiplied by the number of samples was used as the opportunistic infection bacteria expansion score Differences between *MRSA*, *P. aeruginosa* *and S. marcescens* isolates

Opportunistic infection is a condition in which the pathogen does not show pathogenicity in the normal host, but manifests when host resistance is weakened. For opportunistic infection analysis, we assessed 10 types of pathogens: *MRSA*, *MSSA*, *H. influenzae*, *S. agalactiae*, *P. aeruginosa*, *S. pneumoniae*, *K. pneumoniae*, *S. marcescens*, *M. catarrhalis* and *Candida species.* Opportunistic bacterial infections were corrected prior to swabbing and professional care. *MRSA, S. agalactiae *and *S. marcescens* were detected in the oral cavity; however, other bacteria were not isolated. Differences in opportunistic bacteria between the water and MA-T gel groups were compared. The opportunistic bacterial score multiplied by the number of samples denoted the opportunistic infection bacterial expansion score. Data were collected prior to swabbing and professional care. The graph shows the results of the sampling data from three patients three times. Although the bacterial scores tended to decrease with MA-T gel care, no significant difference was observed between water and MA-T gel care (Fig. 5 and Additional file 4: Figure S4).

**Fig. 6:**
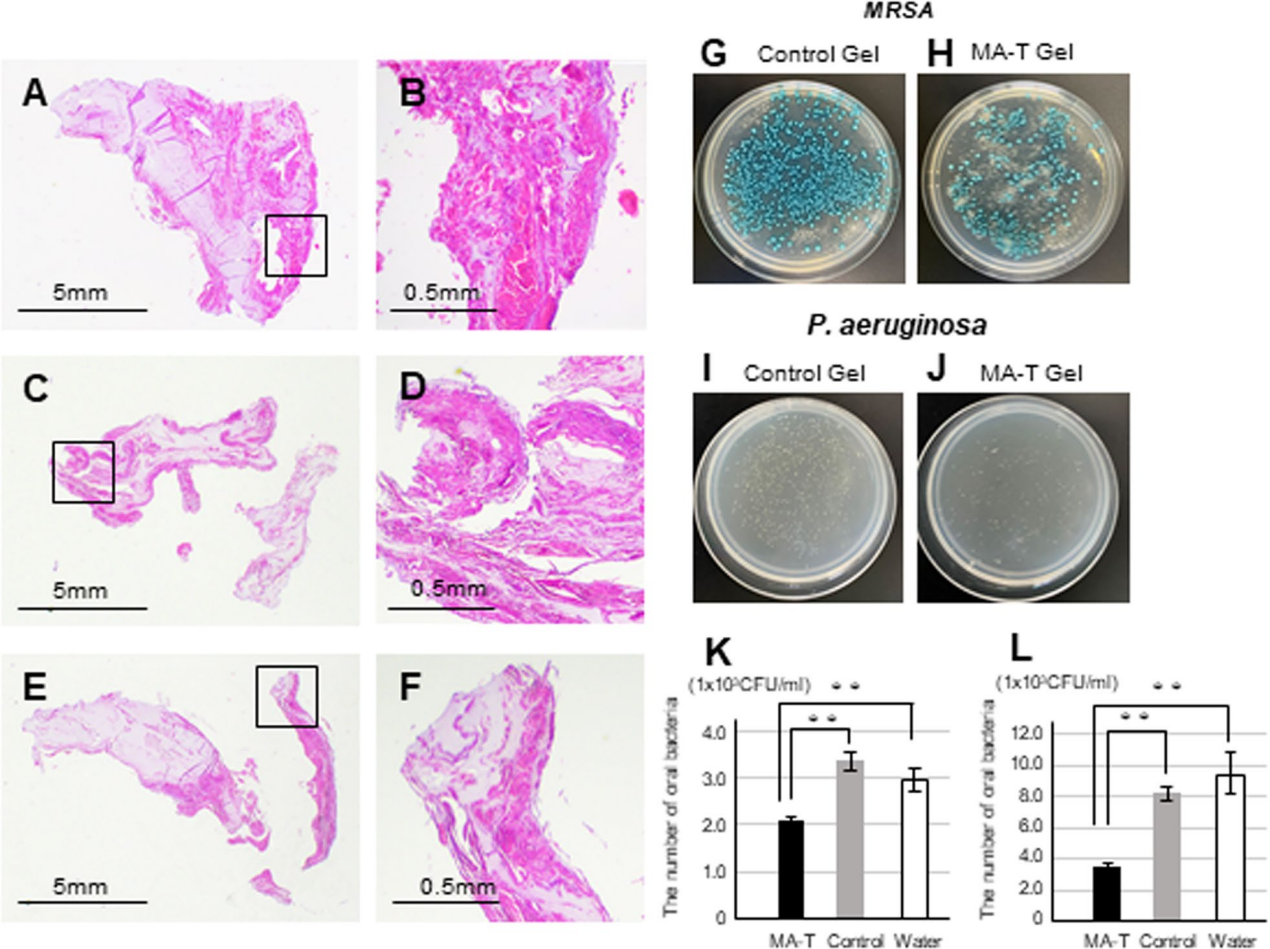
Effect of MA-T gel on opportunistic infection bacteria. Histology of the exfoliated epithelial film from patient A with hematoxylin and eosin staining (**A**, **B**). Patient B (**C**, **D**). Patient C (**E**, **F**). Low-power (**A**, **C**, **E**) and high-power (**B**, **D**, **F**) fields. Differences in colonization between exfoliated epithelial film bacteria and control gel or MA-T gels. The control gel did not contain MA-T. Images of *MRSA* (**G**, **H**) and *P. aeruginosa* infection (**I**, **J**) Colonies. The difference in colony counts in MA-T gel, control gel, and water with *MRSA* (**K**) and *P. aeruginosa* infection (**L**) colonies. **P < 0.01 respectively using the Bonferroni method

Furthermore, we analyzed the laboratory findings to investigate the effects of the MA-T gel. First, histological analysis of the membrane substances was performed. Membranous substances were sampled from two patients. Membranous substances were mainly composed of acidophilic stratified structures and inflammatory cells, such as neutrophils and lymphocytes. The acidophilic stratified elements were squamous epithelium-derived keratinocytes (Fig. 6A–F). The effects of MA-T on these membranes are shown in Fig. 6G–L. The solution was mixed with the bacterial dissolution liquid in half quantity using MA-T gel, control gel and water. The mixed solution was inoculated in the *MRSA* or *S. agalactiae* selective media and cultured. Differences in the number of colonies are shown in Fig. 6I–L. Colony formation was significantly decreased in cells cultured on the MA-T gel. No difference in colony formation was observed between water and control gels. In summary, oral care with MA-T gels decreased opportunistic bacterial infections and maintained oral moisture. The MA-T gel was effective in preventing aspiration-related pneumonia.

## Discussion

None of the patients in this study could take oral feeds and breathing was by mechanical ventilation, due to cerebrovascular disease. High-quality care is necessary because of the difficulties in communicating with such patients. Additionally, daily care should be simple and safe. Oral care must be performed several times a day to protect the oral cavity from bacterial invasion. Although oral care of the elderly is important, physical and mental burdens occur owing to changes in posture and breathing patterns and choking due to aspiration and mental stress [17]. Moreover, aspiration should be prevented during oral care in high-risk patients. Silent aspiration easily occurs when oral hygiene is poor and infected saliva causes aspiration-related pneumonia [18]. Therefore, oral care can prevent aspiration-related pneumonia in older receiving nursing care. Although it is well known that oral care is important, challenges in hospitals and facilities exist because there are fewer staff members. Oral care gels are often used for ease of care and moisturization. This study showed that the use of MA-T gel not only improved moisture retention but also reduced the total bacteria in the oral cavity. Bacterial culture experiments showed that the gel was effective against opportunistic bacteria. This study focused on the membrane substances of high-risk patients who were not taking oral medication. Membranous substances were always detected in patients who did not ingest them orally if their oral cavity were regularly cared for. A previous study showed that membranous substances consist of secretory mucins (Muc5B) from the palatine gland, dried viscous liquid, and bacteria [19]. Tearing a film from the oral mucosa is challenging. The MA-T gel consisted of MA-T and base material. MA-T directly reduced the total bacterial count in the oral cavity by inactivating bacteria, including opportunistic organisms that cause pneumonia. In addition, the base material of the gel was shown to improve the oral humidity. The improvement in intraoral humidity makes it easier to remove dirt, which indirectly reduces the number of intraoral bacteria. Furthermore, the formation of the epithelial film is caused by interference in the turnover of the mucosa [20]. The improvement in oral humidity leads to normal turnover of the oral mucosa and prevents the formation of membrane substances, which are the breeding grounds for bacteria.

Therefore, it is necessary to reduce intraoral bacteria while maintaining high oral humidity. Moreover, oral moisture was high with few bacteria when daily care was continued using the MA-T gel. Our results showed that oral care using MA-T gel was ideal in terms of humidity and hygiene. In this study, we evaluated several bacterial species by using various methods. Many bacteria may exist, even if there is no overt evidence of infection. The medical staff should objectively evaluate the oral cavity of the patient by measuring the number of bacteria. Dentists can recognize changes in general health conditions by evaluating the intraoral state of patients.

It should be noted that MA-T is effective against a variety of bacteria without side effects. This study confirmed the efficacy of the MA-T gel in targeting opportunistic infectious bacteria that cause pneumonia. Pneumonia is the leading cause of death among the elderly [21]. According to data released by the World Health Organization, pneumonia was the third leading cause of death in 2019, causing 2.5 million deaths worldwide [22]. The emergence of coronavirus disease 2019 (COVID-19) caused by severe acute respiratory syndrome coronavirus 2 (SARS-CoV-2) has caused a global pandemic [23]. Pneumonia caused by opportunistic bacteria presents symptoms similar to those caused by SARS-CoV-2 [23]. It is important to provide an agent for the treatment and prevention of fungal infections such as opportunistic infections and SARS-CoV-2. Several transmission routes for SARS-CoV-2 have been proposed, including direct and contact transmission. Importantly, recent studies have proposed that saliva is a potential reservoir for COVID-19, a symptomatic infection [25, 26]. MA-T also has a high bacterial killing ability against SARS-CoV-2 virus [12]. MA-T has the potential to prevent xerostomia with candidiasis and Sjögren syndrome. MA-T gel could be useful for the maintenance of the oral mucosa in patients whose dentures need removal.

The main component of MA-T is chlorine acid. Sodium chlorite is used as a water disinfectant in Europe and the USA as well as in the food industry [27]. In an aqueous solution containing acid (H+), sodium chlorite forms a semi-stable chlorous acid that exhibits disinfectant properties against bacteria and viruses. Although alcohol degrades bacteria, it is difficult to volatilize. Chlorine dioxide is widely used as a disinfectant in laboratories, hospitals, and public spaces because it does not produce toxic by-products unlike chlorine [28]. Noguchi et al. [29] investigated the safety of MA-T ingestion in mice. Mice that were provided drinking water containing 0–3000 μg/mL MA-T for 7 days did not show any changes. Recent studies have indicated the safety of MA-T. This study also demonstrated that MA-T gel is effective and safe for oral care.

Therefore, it is necessary to reduce intraoral bacteria while maintaining high oral humidity. Moreover, oral moisture is high with a few bacteria when daily care was continued using MA-T gel. Our results showed that oral care using the MA-T gel maintains an ideal state in terms of humidity and hygiene. In this study, we evaluated several bacterial species by using various methods. Many bacteria may exist, even if it appears visually clean. It is thought that care from the medical staff should objectively evaluate the oral cavity of the patient by measuring the number of bacteria. Dentists can recognize changes in general health conditions by evaluatively their intraoral state extensively.

This study has some limitations. First, the sample size was extremely small. At the beginning of the study, we tried to recruit more patients; however, some patients could not complete the study because they died. During the COVID-19 pandemic, it was critical to conduct research on high-risk patients. For studies with small sample sizes, cross-sectional studies are common and require examination of the effects of appliances. We had to complete this study within the shortest possible time because high-risk patients were being studied. A 1-month blank was established before the period of professional care with water and MA-T gel. Second, the base material of MA-T was used as the control in the laboratory, but the control gel was not used on the patients to investigate the effect of MA-T. Our ethical committee and patients could not accept that we used unknown gels that are not commercially available for the purpose of oral care. Although water was used as a control in the patient surveys, we used similar quantities of water and the MA-T gel. Glycerin is used for xerostomia because it is included in various oral care gels [30]. Glycerin was also used in cosmetics for skincare products, hair conditioning agents, and body creams [31]. However, glycerin was not used in this study. The molecular weight of glycerin (92.1 g/mol) is higher than water (18.0 g/mol). Glycerin has a high ability to absorb oral moisture; however, it remains in the oral cavity when dried because it has a large molecular weight. Most tube-feeding patients experience oral dryness [32] and glycerin is easily retained in the oral cavity when dried. The MA-T gel did not include Glycerin to keep humidity and is able to avoid membrane substances or oral infection. Thus, MA-T gel could be an ideal product for oral hygiene.

## Conclusions

This study focused on changes in the number of oral bacteria when using MA-T gel and demonstrated the medium-term results of continuing care for 1 month. In the future, it will be necessary to verify the clinical effects of the long-term implementation of using MA-T gel. In this study, we investigated the usefulness of MA-T gels for continuous care. Oral care with MA-T gel improves the oral environment by reducing the number of bacteria and upregulating moisture. Saliva secretion in elderly patients is decreased, and the dry oral cavity could be a reservoir for bacteria causing pneumonia. Therefore, oral care gels with MA-T may prevent opportunistic infections in high-risk patients. MA-T extends healthy life expectancy in high-risk patients.

## Supplementary Information

**Additional file 1**. **Figure S1.** Flow-chart of patient selection. Twenty-eight patients are approached to participate in this study. However, 25 are excluded for several reasons.

**Additional file 2**. **Figure S2.** Flow-chart of the investigation of patients with a single administration. “Single administration” indicates professional care using MA-T gel and daily care with water. The black triangle shows a single administration of professional care using MA-T gel and daily care with water (A). Differences in oral moisture counts between water and MA-T gel care for each patient (B). Normalization data of total bacteria between water and MA-T gel care for each patient. The black triangle shows a single administration of professional care using MA-T gel and daily care with water (C).

**Additional file 3**. **Figure S3**. Differential counts of total bacteria between water and MA-T gel care. Non-normalized data of Figure 3B and App (Figure 2C). The difference in the total number of oral bacteria between water and MA-T gels. These data are sampled from three participants, three times each. *P < 0.05, **P < 0.01 respectively using the Bonferroni method (A). Differences in total bacterial counts between water care and MA-T gel care in each patient. The black triangle shows a single administration of professional care using MA-T gel with daily water care (B).

**Additional file 4**. **Figure S4**. Differences in total bacterial counts between water care and MA-T gel care in each patient. *MRSA* (A), *P. aeruginosa* (B), and *S. pneumoniae* (C) Vertical axis shows opportunistic infection bacterial scores. The opportunistic infection bacteria　expansion score is set as follows: (-): No growth of bacteria, (1+): Bacteria grow up to one-third of the nutrient medium (approximately 1.0×10^3^ CFU/mL), (2+): Bacteria grow up to two-third of the nutrient medium (approximately 1.0×10^4^ CFU/mL), (3+): Bacteria grow to the whole nutrient medium (above 1.0×10^5^ CFU/mL). The large circle indicates that there are many cases.

## Data Availability

The datasets during and/or analyzed during the current study available from the corresponding author on reasonable request.

## References

[1] Clavé P, Shaker R (2015). Dysphagia: current reality and scope of the problem. Nat Rev Gastroenterol Hepatol.

[2] Cabre M, Serra-Prat M, Palomera E, Almirall J, Pallares R, Clavé P (2009). Prevalence and prognostic implications of dysphagia in elderly patients with pneumonia. Age Ageing.

[3] Loeb M, Neupane B, Walter SD, Hanning R, Carusone SC, Lewis D (2009). Environmental risk factors for community-acquired pneumonia hospitalization in older adults. J Am Geriatr Soc.

[4] El-Solh AA, Pietrantoni C, Bhat A, Okada M, Zambon J, Aquilina A (2004). Colonization of dental plaques: a reservoir of respiratory pathogens for hospital-acquired pneumonia in institutionalized elders. Chest.

[5] Kawase Y, Ogasawara T, Kawase S, Wakimoto N, Matsuo K, Shen FC (2014). Factors affecting the formation of membranous substances in the palates of elderly persons requiring nursing care. Gerodontology.

[6] Ghezzi EM (2014). Developing pathways for oral care in elders: evidence-based interventions for dental caries prevention in dentate elders. Gerodontology.

[7] Phuu H, Piedad S, Roseann M (2015). Dry mouth: a critical topic for older adult patients. J Prosthodont Res.

[8] Marcott S, Dewan K, Kwan M, Baik F, Lee YJ, Sirjani D (2020). Where dysphagia begins: polypharmacy and xerostomia. Fed Pract.

[9] Scully C (2003). Drug effects on salivary glands: dry mouth. Oral Dis.

[10] Joanna ND, Thomson WM (2015). Dry mouth—an overview. Singap Dent J.

[11] Urakawa R, Shibata T, Kimura T, Gojo N, Sogou M, Takamori K, et al. The bactericidal effect of oral care using matching transformation system: a prospective clinical study. Oral Sci Int. 2022;1–7.

[12] Shibata T, Konishi K (2020). The respiratory chain of bacteria is a target of the disinfectant MA-T. BPB Rep.

[13] Eilers J, Berger AM, Petersen MC (1988). Development, testing, and application of the oral assessment guide. Oncol Nurs Forum.

[14] Kikutani T, Tamura F, Tashiro H, Yoshida M, Konishi K, Hamada R (2015). Relationship between oral bacteria count and pneumonia onset in elderly nursing home residents. Geriatr Gerontol Int.

[15] Ono Minagi H, Yamanaka Y, Sakai T (2020). Evaluation of the Saxon test for patients with hyposalivation without Sjögren's syndrome. J Oral Rehabil.

[16] Ono H, Obana A, Usami Y, Sakai M, Nohara K, Egusa H, et al. Regenerating salivary glands in the microenvironment of induced pluripotent stem cells. Biomed Res Int. 2015;2015. 10.1155/2015/293570 PMC4491559 26185754

[17] Yokoi Y, Aoki K (1999). Relationship between blood pressure and heart-rate variability during graded head-up tilt. Acta Physiol Scand.

[18] Bassim CW, Gibson G, Ward T, Paphides BM, Denucci DJ (2008). Modification of the risk of mortality from pneumonia with oral hygiene care. J Am Geriatr Soc.

[19] Piras M, Hand AR, Piludu M (2011). Electron microscopic immunogold localization of salivary mucin MUC5B in human buccal and palatal glands. Acta Histochem.

[20] Ogasawara T, Andou N, Kawase S, Kawase Y, Matsuo K, Ozaki Y (2008). Potential factors responsible for dryness of the dorsum of the tongue in elderly requiring care. Gerodontology.

[21] Manabe T, Fujikura Y, Mizukami K, Akatsu H, Kudo K (2019). Pneumonia-associated death in patients with dementia: a systematic review and meta-analysis. PLoS ONE.

[22] Koivula I, Sten M, Makela PH (1994). Risk factors for pneumonia in the elderly. Am J Med.

[23] Bedford J, Enria D, Giesecke J, Heymann DL, Ihekweazu C, Kobinger G (2020). COVID-19: towards controlling of a pandemic. Lancet (Lond Engl).

[24] Liu Y, Lv J, Liu J, Li M, Xie J, Lv Q (2020). Mucus production stimulated by IFN-AhR signaling triggers hypoxia of COVID-19. Cell Res.

[25] Usami Y, Hirose K, Okumura M, Toyosawa S, Sakai T (2021). Brief communication: immunohistochemical detection of ACE2 in human salivary gland. Oral Sci Int.

[26] Xu R, Cui B, Duan X, Zhang P, Zhou X, Yuan Q (2020). Saliva: potential diagnostic value and transmission of 2019-nCoV. Int J Oral Sci.

[27] Malka SK, Park MH (2022). Fresh produce safety and quality: chlorine dioxide’s role. Front Plant Sci.

[28] Praeger U, Herppich WB, Hassenberg K (2018). Aqueous chlorine dioxide treatment of horticultural produce: effects on microbial safety and produce quality–a review. Crit Rev Food Sci Nutr.

[29] Noguchi T, Tachibana K, Inoue T, Sakai T, Tsujikawa K, Fujio Y, Yamagishi Y, Hamaguchi S, Kutsuna S, Kondoh M (2022). Safety evaluation of MA-T after ingestion in mice. Toxicology.

[30] Han P, Suarez-Durall P, Mulligan R (2015). Dry mouth: a critical topic for older adult patients. J Prosthodont Res.

[31] Becker LC, Bergfeld WF, Belsito DV, Hill RA, Klaassen CD, Liebler DC (2019). Safety assessment of glycerin as used in cosmetics. Int J Toxicol.

[32] Padilla GV, Grant M, Wong H, Hansen BW, Hanson RL, Bergstrom N, Kubo WR (1979). Subjective distresses of nasogastric tube feeding. JPEN J Parenter Enteral Nutr.

