# Supplementary figures and images for: Evaluation of oral care using MA-T gel for high-risk patients: a pilot study
Source: BMC Oral Health. 2023 Feb 17;23:108. doi: 10.1186/s12903-023-02779-5 (PMC9936488; doi:10.1186/s12903-023-02779-5)

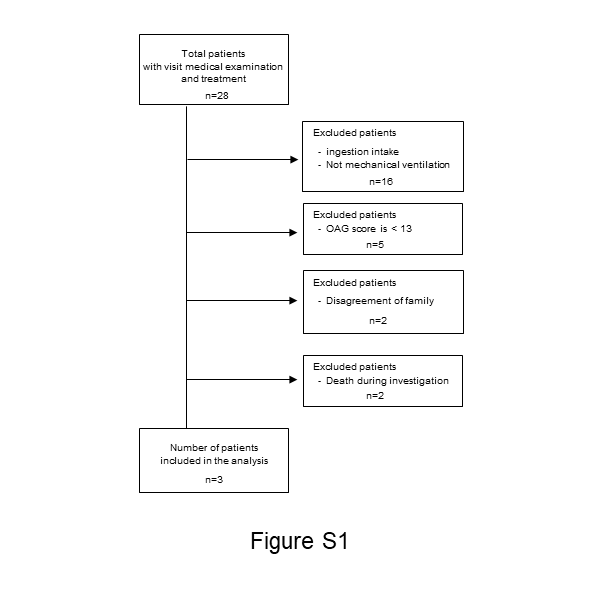

Supplement: Supplementary file 1 — Additional file 1. Figure S1. Flow-chart of patient selection. Twenty-eight patients are approached to participate in this study. However, 25 are excluded for several reasons. [file 12903_2023_2779_MOESM1_ESM.tif]

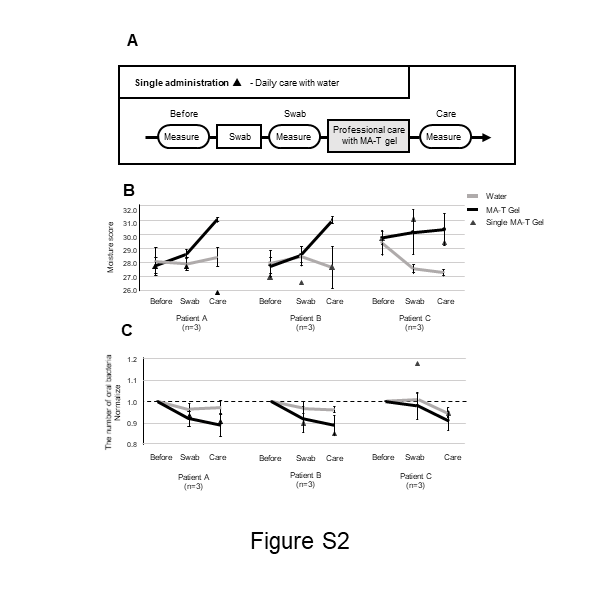

Supplement: Supplementary file 2 — Additional file 2. Figure S2. Flow-chart of the investigation of patients with a single administration. “Single administration” indicates professional care using MA-T gel and daily care with water. The black triangle shows a single administration of professional care using MA-T gel and daily care with water (A). Differences in oral moisture counts between water and MA-T gel care for each patient (B). Normalization data of total bacteria between water and MA-T gel care for each patient. The black triangle shows a single administration of professional care using MA-T gel and daily care with water (C). [file 12903_2023_2779_MOESM2_ESM.tif]

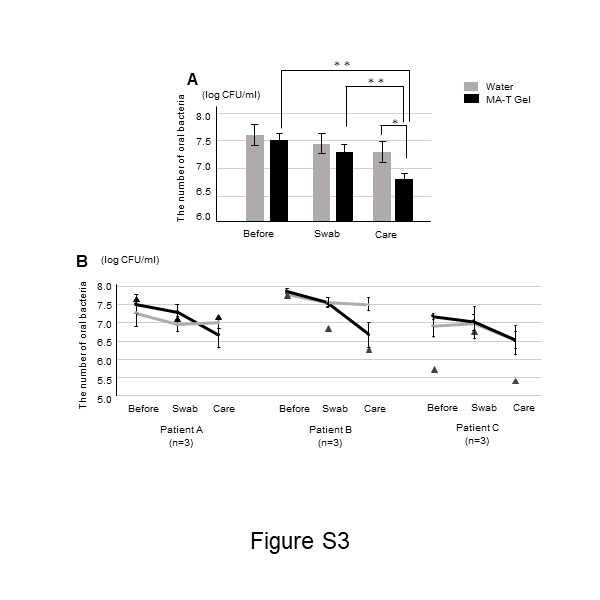

Supplement: Supplementary file 3 — Additional file 3. Figure S3. Differential counts of total bacteria between water and MA-T gel care. Non-normalized data of Figure 3B and App (Figure 2C). The difference in the total number of oral bacteria between water and MA-T gels. These data are sampled from three participants, three times each. *P < 0.05, **P < 0.01 respectively using the Bonferroni method (A). Differences in total bacterial counts between water care and MA-T gel care in each patient. The black triangle shows a single administration of professional care using MA-T gel with daily water care (B). [file 12903_2023_2779_MOESM3_ESM.tif]

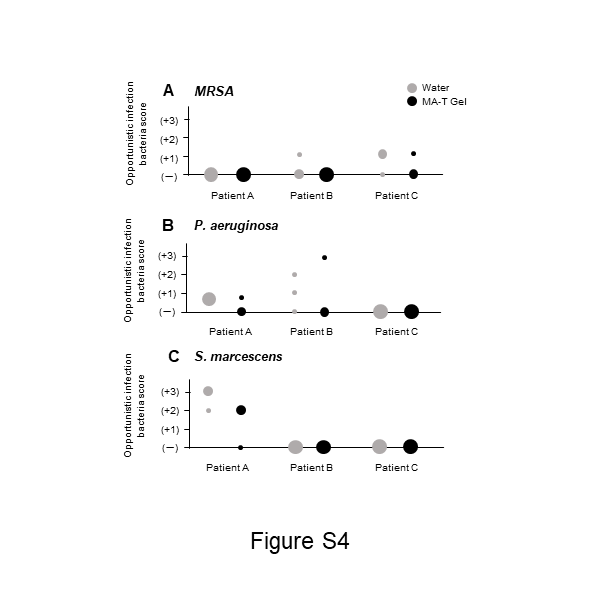

Supplement: Supplementary file 4 — Additional file 4. Figure S4. Differences in total bacterial counts between water care and MA-T gel care in each patient. MRSA (A), P. aeruginosa (B), and S. pneumoniae (C) Vertical axis shows opportunistic infection bacterial scores. The opportunistic infection bacteria expansion score is set as follows: (-): No growth of bacteria, (1+): Bacteria grow up to one-third of the nutrient medium (approximately 1.0×10^3^ CFU/mL), (2+): Bacteria grow up to two-third of the nutrient medium (approximately 1.0×10^4^ CFU/mL), (3+): Bacteria grow to the whole nutrient medium (above 1.0×10^5^ CFU/mL). The large circle indicates that there are many cases. [file 12903_2023_2779_MOESM4_ESM.tif]
